# Moving knowledge about family violence into public health policy and practice: a mixed method study of a deliberative dialogue

**DOI:** 10.1186/s12961-016-0100-9

**Published:** 2016-04-21

**Authors:** Jennifer A. Boyko, Anita Kothari, C. Nadine Wathen

**Affiliations:** School of Health Studies, Faculty of Health Sciences & Faculty of Information & Media Studies, Western University, Health Sciences Building, Room 403, London, ON N6A 5B9 Canada; School of Health Studies, Faculty of Health Sciences, Western University, Health Sciences Building, Room 222, London, ON N6A 5B9 Canada; Health Information Science, Faculty of Information & Media Studies, Western University, London, ON N6A 5B7 Canada

**Keywords:** Knowledge translation, Evidence-informed decision-making, Public health policy, Family violence, Collaborative sensemaking

## Abstract

**Background:**

There is a need to understand scientific evidence in light of the context within which it will be used. Deliberative dialogues are a promising strategy that can be used to meet this evidence interpretation challenge.

**Methods:**

We evaluated a deliberative dialogue held by a transnational violence prevention network. The deliberative dialogue included researchers and knowledge user partners of the Preventing Violence Across the Lifespan (PreVAiL) Research Network and was incorporated into a biennial full-team meeting. The dialogue included pre- and post-meeting activities, as well as deliberations embedded within the meeting agenda. The deliberations included a preparatory plenary session, small group sessions and a synthesizing plenary. The challenge addressed through the process was how to mobilize research to orient health and social service systems to prevent family violence and its consequences. The deliberations focused on the challenge, potential solutions for addressing it and implementation factors. Using a mixed-methods approach, data were collected via questionnaires, meeting minutes, dialogue documents and follow-up telephone interviews.

**Results:**

Forty-four individuals (all known to each other and from diverse professional roles, settings and countries) participated in the deliberative dialogue. Ten of the 12 features of the deliberative dialogue were rated favourably by all respondents. The mean behavioural intention score was 5.7 on a scale from 1 (strongly disagree) to 7 (strongly agree), suggesting that many participants intended to use what they learned in their future decision-making. Interviews provided further insight into what might be done to facilitate the use of research in the violence prevention arena.

**Conclusion:**

Findings suggest that participants will use dialogue learnings to influence practice and policy change. Deliberative dialogues may be a viable strategy for collaborative sensemaking of research related to family violence prevention, and other public health topics.

**Electronic supplementary material:**

The online version of this article (doi:10.1186/s12961-016-0100-9) contains supplementary material, which is available to authorized users.

## Background

Knowledge translation (KT) scholars have promoted the idea that KT, namely the synthesis, exchange, dissemination and implementation of evidence, is a social process dependent on people interacting with one another to situate new knowledge in a specific context [1]. Deliberative dialogues – a group process that emphasizes transformative discussion using synthesized research and contextual knowledge – can help support the use of research evidence by knowledge users through collaborative sensemaking about a pressing health system issue [2–6]. One view of sensemaking is that it refers to a diverse set of KT approaches that support greater understanding and successful implementation of new ideas or interventions in the context of their current understandings [7]. Knowledge users’ understanding of an issue is a key factor in the KT process [8] and a systematic process of making sense of research findings can support implementation [9].

Previous research about deliberative processes for eliciting and combining evidence [2–4] contributed to a model that describes the key features and intended effects of deliberative dialogues as a KT strategy [6]. According to the model, the key features of a deliberative dialogue pertain to (1) the meeting environment (i.e. appropriate resources, skilled facilitation, rules for deliberation); (2) the mix of participants (i.e. ensuring fair representation of relevant interests); and (3) the role of research evidence (i.e. pre-circulating evidence in order to foster similar understanding of the issues among participants) [6]. Recent studies demonstrate that these features can contribute to a successful deliberative dialogue event [10–12]. For example, a case study of a deliberative dialogue that incorporated these key features found that participants perceived them to be useful and concluded that they should be maintained in future dialogues [10].

Studies also highlight other aspects of deliberative dialogues [5, 6, 13]. First, these dialogues can be resource intensive for both the planners and participants [6]. An Australian study concluded that deliberative processes could be used for health policy development, but also found them to be time-consuming and costly [13]. Second, dialogues tend to be performed with knowledge users who are not formally associated with one another. Individuals are typically selected to participate based on their ability to articulate their organization’s views and experiences, engage with others representing different interests and lead future efforts related to addressing the issue at hand [5]. Third, deliberative dialogues are often held in response to a high priority health system challenge. Lavis et al. [5] suggest that policy-focused dialogues should take place when issues “*are considered a high priority and ‘windows of opportunity’ for change are evident*.” While what is known about formative aspects of designing a deliberative dialogue may be helpful, it is not clear how to adapt a deliberative dialogue in light of their potential challenges, and how outcomes will be affected by these adaptations.

The purpose of the present study was to explore the features and outcomes of a deliberative dialogue characterized as (1) embedded into an existing meeting agenda as a way to maximize resources; (2) including participants belonging to a mature network, and who therefore had previous interactions; and (3) part of a planned KT strategy to mobilize research produced by the network. The network was established in 2009 and has since continued to mature by supporting an integrated research and KT agenda that includes specific efforts (such as the deliberative dialogue described in this paper) aimed at supporting the spread and uptake of the research produced through the network [14]. An evaluation of the network during its formative stage found that members valued the network in terms of, for example, supporting their professional and organizational mandates [14].

The topic under deliberation – preventing violence against women and children and its consequences, across the lifespan, while emphasizing the role of resilience factors – met the criteria of timeliness and priority. Specifically, family violence, and the intersection of violence and trauma with other social factors, is recognized as a pressing social determinant of health. WHO’s ecological framework and public health approach to violence prevention situates violence as a fundamental public health concern and outlines that its causes, consequences and intervention points occur at the individual, family, community and societal levels [15]. Current priorities in Canada, the United States of America and beyond [16], including multi-lateral efforts by WHO (e.g. through successful enactment of the World Health Assembly’s historic resolution on “*strengthening the role of the health system in addressing violence, in particular against women and girls, and against children*” in May 2014), as well as recent high-profile attention to the issue of family violence, underscore the timeliness of the dialogue.

## Methods

### Design

The research questions for this study were (1) How are formative aspects related to key design features of the deliberative dialogue viewed by participants? (2) How do participants intend and actually use the knowledge arising from the deliberative dialogue? (3) How might deliberative dialogues be tailored to support the uptake of violence prevention research? A mixed methods approach that included questionnaires and semi-structured interviews was used to create a comprehensive account of the deliberative dialogue that combines contextual understanding with what is currently known about key features and effects of deliberative dialogues [17].

### Participants

Dialogue participants included attendees of the Preventing Violence Across the Lifespan (PreVAiL) [18] Research Network’s biennial full team meeting. PreVAiL has more than 40 researchers and 20 knowledge user partners (i.e. from national and supranational organizations) and its mandate is to develop and mobilize research about child maltreatment and intimate partner violence (i.e. family violence), with particular focus on resilience factors. The knowledge users included a range of policy and practice actors with responsibilities related to mental health, gender, and/or violence from organizations (governmental, NGOs) with service, research and policy mandates in these areas. Individuals who participated in the deliberative dialogue, as well as individuals involved in planning, implementation and evaluation (i.e. steering and working groups), were invited to participate in the study.

### Deliberative dialogue process

**Fig. 1:**
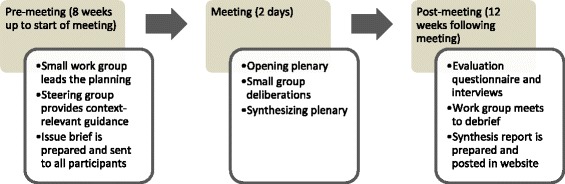
Summary of PreVAiL deliberative dialogue structure and process

The deliberative dialogue structure and process is outlined in Fig. 1. The PreVAiL co-leads convened a small planning workgroup that included three PreVAiL members as well as staff and trainees who provided process-related support and deliberative dialogue expertise. The workgroup convened a steering committee of five PreVAiL partners (all policy actors) to provide context-relevant guidance, including identifying the overall challenge and priority issues to be addressed. The groups met via teleconference and collaborated via email. It was decided that the dialogue would address the overall challenge of how to use research evidence to orient health and social systems to prevent child maltreatment and intimate partner violence and their consequences. Given the broad scope of this challenge, three high priority aspects of it were focused on (1) re-orienting existing government violence prevention policy frameworks towards socio-ecological approaches and responses based on rigorous intervention research; (2) building capacity to harness existing research and knowledge about factors related to preventing violence; and (3) engaging stakeholders and the broader public in developing principles and strategies for communicating evidence-based violence prevention messages.

The work and steering groups collaborated via teleconference and email to compile an issue brief detailing the challenge, the priority issues and relevant research evidence, and to plan logistics of the dialogue process at the meeting. In order to fit within the meeting agenda, it was decided to include a preparatory plenary session, concurrent sessions that each focused on a different priority issue, and a final synthesizing plenary session. Attendees, who received the issue brief a week in advance of the meeting, were asked to select and participate in the concurrent session most closely aligned with their field of research or practice. During each concurrent session of 10–15 participants, the deliberations addressed (1) the problem; (2) potential solutions for addressing it; and (3) factors (specific and global) to consider in terms of implementing solutions (Fig. 1). The deliberations also incorporated a number of key design elements that have been shown to be helpful in deliberative dialogues (Table 1). The overall planning process took approximately 6 weeks and the deliberative dialogue was held over 2 days.

**Table 1:** Ratings of key design features

Design features^a^	Role categories		
	All (n = 22)^b,c^	Researchers (n = 12)^d^	Partners (n = 8)^e^
	Mean (standard deviation)		
Addressed high-priority policy issues	5.2 (1.5)	5.4 (0.7)	5.3 (1.4)
Provided an opportunity to discuss different aspects of the issues	5.5 (1.5)	5.6 (0.8)	5.3 (1.4)
Provided an opportunity to discuss possible options for addressing issues	5.2 (1.5)	5.2 (1.0)	5.6 (1.2)
Provided an opportunity to discuss key implementation considerations	5.0 (1.5)	4.9 (0.7)	5.3 (1.2)
Provided an opportunity to discuss who might do what differently	4.9 (1.3)	4.5 (1.3)	5.2 (0.9)
Deliberative was informed by a pre-circulated issue brief	5.2 (1.6)	5.5 (1.2)	5.8 (1.6)
Included discussion about factors that can inform how to approach the issues, possible options for addressing them and key implementation considerations	5.4 (1.5)	5.2 (1.6)	5.6 (1.0)
Brought together parties (including researchers and knowledge user partners) who could be involved in or affected by decisions related to the issues	6.1 (1.4)	6.0 (1.4)	6.2 (1.2)
Was limited to PreVAiL researchers and partners	5.7 (1.4)	5.8 (0.8)	5.6 (1.2)
Engaged a facilitator to assist with the deliberations	6.0 (1.4)	5.5 (1.7)	6.5 (0.3)
Did not aim for consensus	5.5 (1.4)	5.2 (1.4)	6.4 (0.7)
Allowed for frank, off-the-record deliberations following the Chatham House Rule	6.3 (1.1)	6.1 (1.2)	6.7 (0.6)

^a^Questions pertaining to design features were on a scale from 1 (very unhelpful) to 7 (very helpful)

^b^The number of participants who responded to each question was 21 or 22

^c^Three participants did not provide responses to their role categories and one participant identified as both researcher and partner

^d^The number of researchers who responded to each question was 11 or 12

^e^The number of partners who responded to each question was 7 or 8

### Data collection

#### Questionnaire

A previously developed questionnaire [19] was used to assess the deliberative dialogue in terms of formative and summative aspects. Minor changes were made to the questionnaire in order to make it contextually relevant to those completing it. For example, we changed the term ‘stakeholder dialogue’ to ‘deliberative dialogue’ and the term ‘evidence brief’ to ‘issue brief’. Modifications were also made to the section pertaining to demographic information. Prior to using the adapted version of the questionnaire the workgroup and steering committee reviewed it for face validity. Although we did not formally pilot test the questionnaire, the workgroup and steering committee were representative of those who would be using it. The final questionnaire included five sections: (1) 12 formative evaluation questions related to how useful participants found specific features of the dialogue (i.e. those in Table 1); (2) one question focused on an overall assessment of the deliberative dialogue; (3) four questions focused on views about future deliberative dialogues; (4) 12 summative evaluation questions related to intention to use what was discussed at the dialogue in their future decision-making; and (5) four questions related to role and background. Each formative and overall assessment question included a 7-point Likert-type scale and space for written responses, whereas the summative questions included only 7-point Likert-type scales. Previous research demonstrates that the summative evaluation questions, based on the theory of planned behaviour that posits that ‘intention’ is an immediate precursor of actual behaviour, have good internal consistency and test-retest reliability [19, 20]. Three evaluation questions measured the strength of participants’ intention to use the research discussed at the dialogue. Similar to other studies, we considered these questions to be a measure of the deliberative dialogue’s short-term impact [11, 12]. Dialogue participants were asked to complete the questionnaire at the meeting immediately following its final session. Further details about the questionnaire can be obtained from the corresponding author.

#### Semi-structured interviews

From 1 to 3 months after the dialogue, participants were invited to take part in a follow-up telephone interview in order to explore (1) the overall deliberative dialogue experience; (2) the usefulness of specific aspects of the deliberative dialogue; and (3) themes emerging from the deliberative dialogue, as well as preliminary analysis of the formative evaluations. The interview questions are included in Additional file 1.

#### De-briefing

The deliberative dialogue workgroup held a de-briefing session immediately following the event and again 2–3 weeks later to explore their own views and experiences regarding the formative aspects of the dialogue.

### Data analysis

The interviews and debrief sessions were audio recorded and transcribed. We analyzed the questionnaire data using descriptive statistics. The open-ended responses from the questionnaires and interview transcripts were coded using nVivo 10 software according to a pre-determined framework based on the questionnaire. The research team then met to discuss and compare the quantitative and qualitative data, and identify areas of consistency and dissonance. Meeting minutes and documents produced as part of the dialogue were used to understand the overall deliberative dialogue process and structure.

### Ethical considerations

The study protocol was approved by Western University Research Ethics Board (Protocol#: 105300). All attendees of PreVAiL’s biennial full team meeting provided consent to participate in this study.

## Results

A total of 44 individuals participated in the deliberative dialogue and the questionnaire response rate was 50% (n = 22). Respondents included PreVAiL researchers (n = 12), knowledge user partners (n = 8), one respondent self-identified as both a researcher and policy actor, and three (n = 3) did not provide a response. Overall, 26 (n = 26) individuals participated in a follow-up interview (63%), including 13 researchers and 13 knowledge user partners.

### Overall assessment

The mean overall assessment of the deliberative dialogue (in response to the question “How well did the deliberative dialogue achieve its purpose?”) was 5.3 on a scale from 1 (failed) to 7 (achieved), with a mean score of 5.3 (standard deviation (SD), 1.4) for researchers and 5.4 (SD, 0.7) for partners. Written comments reflected that participants thought the deliberative dialogue was informative and interesting, but could have been improved. For example, one participant noted that: “*the meeting was informative, but there is a limit to how much can be achieved in the time available*” (PreVAiL Partner). Another participant commented: “*I think we got lost a bit in the last part*” (PreVAiL Partner). Findings from the interviews reflected that participants felt a sense of ‘vagueness’ about the intended goals of the deliberative dialogue. However, there were also many comments that reflected an overall positive experience:

“*It was a change of pace from the other parts of the meeting, which was I think a good thing to do. Too often you, at the meeting, you just sit and listen to talk after talk after talk, and this was a way of making everybody get up and move to another location and I thought it kind of put people into the discussions themselves, and I just thought that was really quite effective in the pattern of the meeting*.” (PreVAiL Partner)

“*I found it very interesting. I think that materials beforehand and being able to discuss it with people who have a lot of expertise was very helpful for me, I learned a lot during the process and also because I was aware of all the materials beforehand I had some time to reflect so I think it was a very successful use of time*.” (PreVAiL Partner)

Overall, the questionnaire and interview findings suggest that participants found the deliberative dialogue to be a worthwhile experience despite room for improvement.

### Views about how the deliberative dialogue was designed

Table 1 describes the participants’ ratings of their views about the 12 design features evaluated. Generally, the participants rated all the design features favourably (5.0 or higher on a 7-point scale of useful to not) with the exception of “provided an opportunity to discuss who might do what differently” which had a mean score of 4.9. The highest rated design feature (“allowed for frank, off-the-record deliberations following the Chatham House Rule”) and the lowest rated feature (“provided an opportunity to discuss who might do what differently”) were the same among researchers and knowledge user partners. According to the Chatham House Rule, participants are free to use information they learned at a meeting, but cannot identify the speaker or any other meeting participant [21].

The biggest difference in mean scores between researchers and knowledge user partners was “did not aim for consensus” suggesting that partners may value this design element more than researchers. This finding was consistent with the interview data. For example, a researcher commented that:

“*I think the saying that we’re not aiming for consensus is, I think that on balance is a negative because it means people feel they don’t have to, if they’ve got an opinion they don’t have to strive to be convincing. I’m not quite sure, when you say we’re not aiming for consensus, we need consensus to do something, I mean we don’t have to have complete agreement, but we need to have I would have thought consensus on what it’s worth agreeing on, about what is possible to agree about, what’s worth doing*.” (PreVAiL Researcher)

In contrast, a knowledge user partner commented that:

“*Personally, I liked that we don’t aim for consensus, I just, I think consensus is, I think the best form of consensus, if you actually have to come to a decision point, then the best form of consensus from my perspective is what can you live with, but, but if you’re not seeking it, if you’re not trying to get to a decision point, then I’m not so sure it’s absolutely critical to have consensus so I’m good with that*.” (PreVAiL Partner)

The interview data provided additional insight about the design elements not captured as part of the questionnaire. For example, while several participants commented that they thought the dialogue “brought together all parties who could be affected by the outcome” a few participants noted a lack of representation. For example:

“*We were talking a lot about minority women, women of color, and things like that and then me and a colleague looked around at the end of it, we looked at each other and we were like you know, we’re sitting here talking about this stuff and there is not one woman of color or man of color in this group and so we realized that we were really not diverse, we were diverse in the jobs that people had and the professions and the experience and expertise that they brought to the table, but racially and ethnically diverse; we were not*.” (PreVAiL Researcher)

Interview data also reflected mixed opinions about the item “engaged one or more skilled facilitators to assist with the deliberations.” While several comments indicated that having a facilitator was very helpful, other comments reflected that the concurrent sessions required strengthened facilitation. One researcher participant suggested “*a co-chair who is an expert in the topic area*” who could “*really pick up and push along the practice points*” (PreVAiL Researcher). On the other hand, the issue brief was clearly perceived as a useful input into the deliberative dialogue and the participants did not note any substantial areas for improvement. Generally, the interview data suggest that all design elements were perceived as useful, with some participants providing constructive input with regards to a few elements.

Written comments and interview data also provided insight about the design features to maintain, retain and change in future dialogues. Generally speaking, the aspects of the dialogue that should be retained included the issue brief, Chatham House Rule, facilitators to assist with deliberations and bringing PreVAiL researchers and partners together in small groups for deliberations. Participants also seemed to like the overall format of the deliberative dialogue taking place over 2 days with small concurrent sessions and plenaries. At least eight participants provided written comments that suggested that the most important design feature to change for future dialogues is the focus of the questions and discussions. One participant noted that “*topics and questions are key*” (PreVAiL Researcher) and another suggested to “*scale down the questions*” noting that the “*the issues at hand were so large and complex*” (PreVAiL Researcher). Similarly, a researcher participant noted during an interview that “*the questions were a little bit too big to tackle in such a short amount of time*”.

### Potential actions arising from the dialogue

The questionnaire asked participants to identify key learnings in terms of actions that PreVAiL as whole, or themselves as individuals in their own roles, can do better or differently to address the priority issues. Comments made by participants suggest that PreVAiL could work towards building a common frame of reference across the different forms of violence addressed by the Network, focus on strengthening partnerships with government actors, develop capacity by partnering with researchers from low- and middle-income countries, and continue supporting mentorship or trainee programs. Key themes among comments about what participants could personally do better or differently to address the key issues included dissemination of knowledge generated by PreVAiL researchers and partners, enhancing their networks through increased collaboration and building and maintaining these contacts, and developing a better understanding of the context and theory surrounding key debates. These potential actions arising from the dialogue suggest that participants did take away some key learning in regards to the overall challenge and priority issues. Further, the richness of responses during the interviews suggests that participants were quite engaged with the process.

### Intended and actual use of the knowledge arising from the dialogue

The mean score of the three items that measured intention to use research evidence arising from the dialogue was 5.5 on a scale from 1 (strongly disagree) to 7 (strongly agree). The interviews provided some insight into the use of knowledge arising from the dialogue (i.e. short-term impact). For example, three participants indicated that they had already used what was learned at the deliberative dialogue:

“*One thing I thought was really interesting that I did learn and I’ve already actually applied is the idea of knowledge translation for media messaging and that I do sometimes speak with the media and I try to have my sound bite and get my message across, but I wasn’t actually aware there was literature and information on how to formally develop these messages and to do it in a knowledge translation framework, so that’s something I thought was definitely interesting and I definitely learned from the dialogue*.” (PreVAiL Researcher)

“*I shared all of the written materials that were circulated before, and indeed, materials I collected also at the meeting. I briefly spoke to my staff in fact about my experience at this event, not in any detailed way, but I spoke about this experience and certainly told them how personally I felt it had been a useful experience for me*.” (PreVAiL Partner)

“*I remember when I came back just being able to go to my colleagues … we talked about building awareness and from a researcher’s perspective there’s lots of concerns about unintended consequences, about the readiness of the service, state of service provision and provinces and so I did come out with some of what I wanted*.” (PreVAiL Partner)

While there were limited examples drawn from the interviews that demonstrate short-term impact, some key themes that emerged were that participants learned about research evidence pertaining to the issues and that coming together to discuss the evidence, where multiple (though still somewhat limited) perspectives were brought forward, contributed to new ideas and insight that they could use moving forward.

## Discussion

In order to answer our research questions we studied a deliberative dialogue held as part of PreVAiL’s biennial team meeting. The challenge and issues addressed by the dialogue were identified and framed with the assistance of a steering group of policy actors representing national and international public health organizations and broadly related to transforming systems to prevent family violence. Using the knowledge base that exists regarding deliberative dialogue interventions, we planned, implemented and evaluated a dialogue. This process was informed from a theoretical perspective by a framework that describes deliberative dialogues as a strategy for system-level KT [6].

Our findings suggest that deliberative dialogues can be successfully adapted to suit an international research meeting that includes a predetermined group of participants with common interest and motivation. Consistent with other studies [10–12], success was reflected by the perceived usefulness of the design features examined, as well as the intention to use what was learned by dialogue participants. Although the deliberative dialogue was generally perceived as successful, our study also provides new insight into a few specific features which can be used to improve upon dialogue processes in similar contexts.

First, it is important that the question to be addressed is focused and clear in scope. Our findings suggest that one or more of the priority aspects of the challenge addressed through the deliberative dialogue process may have been too broad. However, it is not clear which ones since our relatively small sample precluded meaningful sub-group analysis. Although general guidance exists to assist deliberative dialogue planners in appropriately defining the question or problem to be addressed [5], such guidance does not suggest ways to frame questions and problems for deliberative dialogues in particular; what counts as narrow or focused in one context may not in another. While we used a steering group to assist in making these decisions, perhaps broader consultation with participants (or some other form of pilot-testing questions) may have assisted with this task.

Second, planners should consider facilitation supports that enhance deliberation among group members known to each other. Facilitation is a key design feature of deliberative dialogues [2, 5, 6], yet little is known about the characteristics of facilitation or facilitators required when individuals are known to each other. The broader facilitation literature may provide useful guidance in this regard. For example, research about the characteristics of health promotion group facilitators [22, 23] may be transferrable to deliberative dialogues that aim to support the uptake of research into practice. In our case, and as part of PreVAiL’s commitment to trainee development opportunities, we used relatively inexperienced small group facilitators (doctoral and post-doctoral trainees) with varying experience in the violence area. Perhaps more seasoned and/or content-expert co-facilitators are warranted, especially for complex topics.

Third, it is important to frame outputs of the dialogue so that participants understand the goals and feel their efforts have contributed to a tangible outcome. Participant commitment is a key aspect of deliberative dialogues that can be fostered if participants feel they will benefit and gain new thinking and approaches to solving the problem. Thus, to avoid participant disappointment, thought must be taken to ensure the intended effects of a deliberative dialogue are clear from the outset. The effects can be at the short-term individual level (e.g. fostered sense of empowerment); at the medium-term organizational/community level (spinoff partnerships created at the dialogue); or at the long-term system level (e.g. evidence-informed decision-making by policymakers) [6]. Clarifying the outcomes of a dialogue will ensure participants are not left feeling like goals were unclear or not achieved.

Fourth, while topics addressed by deliberative dialogues should relate to pressing health system needs, our study reveals that these can reflect a more global health or social problem. The challenge addressed by the dialogue we studied – family violence prevention – is a challenge currently being addressed by health and social systems worldwide, but also by those in other contexts, including justice and labour, amongst others. Planners and researchers can use the insights we gleaned from our study about design features to improve their approach and the resulting outcomes.

There is a growing knowledge base about the effectiveness of KT interventions that aim to improve professional practice by identifying and proactively taking into account barriers to change [24]. For example, we know that tailored interventions are more likely to improve clinical professional practice compared to having no intervention [25]. However, less is known about how to tailor KT interventions targeting health system decision-makers (e.g. policymakers, senior managers) focused on supporting system-level change. In the context of public health, a randomized controlled trial did find that tailored messages plus access to an online registry of systematic reviews showed a significant positive effect on public health policies and programs [26]. Another review found that prospects for research use in policy improved with increased interactions between researchers and policymakers (e.g. networks) and when research matched the beliefs, values, interests or political goals and strategies of policy actors (e.g. elected officials, advocacy groups) [27]; however, when these beliefs and values are mismatched, including in the family violence area, the uptake of evidence becomes far less predictable [28]. These two prospects for change were met in our evaluation study, and we were able to tailor the KT strategy to suit a particular audience, namely a transnational violence prevention network. Thus, while it may be feasible to tailor system-level KT strategies based on available research guidance, it is not clear whether the approach to tailoring is effective [29]. Our findings do suggest there is some potential impact; however, future research in this area must continue to evaluate KT effects (including benefits, harms and costs [30]) to determine whether efforts to tailor KT strategies are worthwhile.

The research literature about sensemaking provides some useful insight into planning and evaluating system-level KT strategies focused on evidence interpretation. Maitlis and Christianson define sensemaking as a “*process through which people work to understand issues or events that are novel, ambiguous, confusing, or in some other way violate expectations*” [31] (p. 57). In line with deliberative dialogues, this process is characterized by having a safe environment to discuss issues as well as solutions that combine research evidence with practical issues [9]. The sensemaking process can be supported by tools to help identify and assess relevant information, and make collaborative decisions informed by facts, context and political or social sensitivities. For example, a group of researchers developed the Tool for Evaluating Research Implementation Challenges (TECH), which can be used by research teams involved in complex implementation settings to systematically define challenges, assess their potential impact on intervention fidelity and determine next actions [9]. Although further research and evaluation of TECH is required, the pilot study concluded that TECH is an appropriate tool for use in complex settings such as nursing homes or hospitals [9]. This study contributes to the growing literature about sensemaking, with a specific focus on evaluating the impact of KT interventions that aim to support system-level scientific evidence interpretation for subsequent policy and practice decision-making.

### Limitations

Our study findings should be considered in light of limitations of the research. One limitation of our study is the specificity of context (i.e. the deliberative dialogue included a particular content-focused group that were known to each other through a network), which means our findings might only be transferrable to similar situations. A second is that our study involved one deliberative dialogue conducted among a relatively small group, which did not allow for statistical comparisons between different types of participants, only potential trends. Studying a series of contextually similar deliberative dialogues, and aggregating findings across larger samples, might yield a larger pool of participants with more heterogeneity, which might improve the generalizability of questionnaire results. We also conducted the follow-up interviews after a relatively short timeframe, limiting our ability to fully assess the extent to which information gained at the dialogue was used. Nevertheless, there is also a need for more understanding about how to assess the longer-term impacts arising from deliberative dialogues, including influence on policy processes. Relatedly, the tool we used to assess the deliberative dialogue for its formative and summative aspects has a moderate research base supporting its validity or reliability. While this may be the result of deliberative dialogues being an understudied KT intervention, it is important to consider that other approaches to measurement (e.g. line scales, 9-point scales) may yield more precise results or that other approaches to evaluation (e.g. purely qualitatively) may yield more contextually meaningful results.

## Conclusion

Our findings shed light on how deliberative dialogues used as a KT strategy can move forward the research produced by researcher–policymaker networks, such as PreVAiL, by creating a space for structured and frank sensemaking discussion that could lead to a more realistic understanding of how to affect positive system changes for children, women and men exposed to child maltreatment and intimate partner violence. Research and other networks that are interested in using a deliberative dialogue approach should consider the lessons learned from our study. Researchers in the violence prevention arena might consider exploring innovative ways, such as deliberative dialogues, to move their research into action, and KT researchers should continue evaluating outcomes arising from dialogues and other tailored KT sensemaking strategies to determine more long-term impacts on policy and practice processes.

## Additional file

Additional file 1: Deliberative dialogue interview questions. (DOCX 19 kb)

## Abbreviations

KT: Knowledge translation
PreVAiL: Preventing violence across the lifespan
