# Supplementary material for: Moving knowledge about family violence into public health policy and practice: a mixed method study of a deliberative dialogue
Source: Health Res Policy Syst. 2016 Apr 21;14:31. doi: 10.1186/s12961-016-0100-9 (PMC4839163; doi:10.1186/s12961-016-0100-9)
Supplement: Additional file 1: — Deliberative dialogue interview questions. (DOCX 19 kb) [file 12961_2016_100_MOESM1_ESM.docx]

**Deliberative Dialogue Interview Questions**

1. Overall experience at the deliberative dialogue.
   1. How would you describe your experience at the deliberative dialogue?
   2. What was the best thing about the deliberative dialogue?
   3. What was the worst thing about the deliberative dialogue?
   4. Tell me about one thing you learned from participating?
   5. Can you also tell me about one thing you had hoped to learn from participating, but did not?
2. Usefulness of specific aspects of the deliberative dialogue.
   1. Which elements do you feel were most helpful? Can you tell me about why you feel ____ was useful?
   2. Which elements do you feel were least helpful? Can you tell me about why you feel ____ was not useful?
3. Themes that emerged from the deliberative dialogue and the questionnaire.
   1. Do you feel the role of PreVAiL was adequately addressed in thinking about moving issues forward? Please tell us more about this.
   2. How useful were the pre-circulated materials to you? Is there anything you would change about them or the process of receiving them?
   3. Did you feel the balance of discussion between the high-level complexity of an issue and on-the-ground “solutions” or go-forward strategies was achieved? Why or why not?
